# Large Scale Full-Length cDNA Sequencing Reveals a Unique Genomic Landscape in a Lepidopteran Model Insect, *Bombyx mori*

**DOI:** 10.1534/g3.113.006239

**Published:** 2013-09-01

**Authors:** Yoshitaka Suetsugu, Ryo Futahashi, Hiroyuki Kanamori, Keiko Kadono-Okuda, Shun-ichi Sasanuma, Junko Narukawa, Masahiro Ajimura, Akiya Jouraku, Nobukazu Namiki, Michihiko Shimomura, Hideki Sezutsu, Mizuko Osanai-Futahashi, Masataka G Suzuki, Takaaki Daimon, Tetsuro Shinoda, Kiyoko Taniai, Kiyoshi Asaoka, Ryusuke Niwa, Shinpei Kawaoka, Susumu Katsuma, Toshiki Tamura, Hiroaki Noda, Masahiro Kasahara, Sumio Sugano, Yutaka Suzuki, Haruhiko Fujiwara, Hiroshi Kataoka, Kallare P. Arunkumar, Archana Tomar, Javaregowda Nagaraju, Marian R. Goldsmith, Qili Feng, Qingyou Xia, Kimiko Yamamoto, Toru Shimada, Kazuei Mita

**Affiliations:** * National Institute of Agrobiological Sciences, Tsukuba 305-8634, Japan; † National Institute of Advanced Industrial Science and Technology, Tsukuba 305-8566, Japan; ‡ State Key Laboratory of Silkworm Genome Biology, Southwest University, Chongqing 400716, China; § Mitsubishi Space Software Co., Ltd., Tsukuba 305-8602, Japan; ** Department of Integrated Biosciences, Graduate School of Frontier Sciences, The University of Tokyo, Kashiwa 277-8562, Japan; †† Faculty of Life and Environmental Sciences, University of Tsukuba, Tsukuba 305-8572, Japan; ‡‡ Department of Agricultural and Environmental Biology, Graduate School of Agricultural and Life Sciences, The University of Tokyo, Tokyo 113-8657, Japan; §§ Department of Computational Biology, Graduate School of Frontier Sciences, The University of Tokyo, Kashiwa 277-0882, Japan; *** Human Genome Center, Institute of Medical Science, The University of Tokyo, Tokyo 108-8639, Japan; ††† Laboratory of Molecular Genetics, Centre for DNA Fingerprinting and Diagnostics, Hyderabad 500001, India; ‡‡‡ The University of Rhode Island, Kingston, Rhode Island 02881; §§§ Guangdong Provincial Key Laboratory of Biotechnology for Plant Development, School of Life Sciences, South China Normal University, Guangzhou 510631, China

**Keywords:** *Bombyx mori*, large-scale full-length cDNA collection, tissue-specific genes, sexual dimorphism, gene cluster, silkworm

## Abstract

The establishment of a complete genomic sequence of silkworm, the model species of Lepidoptera, laid a foundation for its functional genomics. A more complete annotation of the genome will benefit functional and comparative studies and accelerate extensive industrial applications for this insect. To realize these goals, we embarked upon a large-scale full-length cDNA collection from 21 full-length cDNA libraries derived from 14 tissues of the domesticated silkworm and performed full sequencing by primer walking for 11,104 full-length cDNAs. The large average intron size was 1904 bp, resulting from a high accumulation of transposons. Using gene models predicted by GLEAN and published mRNAs, we identified 16,823 gene loci on the silkworm genome assembly. Orthology analysis of 153 species, including 11 insects, revealed that among three Lepidoptera including Monarch and Heliconius butterflies, the 403 largest silkworm-specific genes were composed mainly of protective immunity, hormone-related, and characteristic structural proteins. Analysis of testis-/ovary-specific genes revealed distinctive features of sexual dimorphism, including depletion of ovary-specific genes on the Z chromosome in contrast to an enrichment of testis-specific genes. More than 40% of genes expressed in specific tissues mapped in tissue-specific chromosomal clusters. The newly obtained FL-cDNA sequences enabled us to annotate the genome of this lepidopteran model insect more accurately, enhancing genomic and functional studies of Lepidoptera and comparative analyses with other insect orders, and yielding new insights into the evolution and organization of lepidopteran-specific genes.

The domesticated silkworm, *Bombyx mori*, is renowned for silk production as well as being a traditional model insect. As the first animal to demonstrate the application of Mendel’s laws (Toyama 1906), it has served as a subject for genetic, physiologic, and developmental studies. A high-quality genome sequence, “scaffold build2” (The International Silkworm Genome Consortium 2008), combined with comprehensive map information (Yamamoto *et al.* 2008), transcriptome analyses (Mita *et al.* 2003; Xia *et al.* 2007), and transgenic technology (Tamura *et al.* 2000; Uchino *et al.* 2008), has enabled dramatic progress in silkworm studies, including positional cloning of mutations (*e.g.*, Ito *et al.* 2008; Liu *et al.* 2010) and genomics of other insects, especially Lepidoptera (Beldade *et al.* 2009; Gahan *et al.* 2010; van’t Hof *et al.* 2011), which were integrated into KAIKObase (http://sgp.dna.affrc.go.jp/KAIKObase/) and SilkDB (http://silkworm.swu.edu.cn/silkdb/).

Lepidoptera is the second-largest order of insects and includes many biologically and economically beneficial species as well as some of the most global and agriculturally destructive pests. Information from the *Bombyx* genome has served as a critical reference for studies of other lepidopterans, and recent reports of chromosomal synteny conservation, even between members of different superfamilies (van’t Hof *et al.* 2011; d’Alençon *et al.* 2010; Zhan *et al.* 2011; The Heliconius Genome Consortium 2012), illustrate the value of the *B. mori* genome as a model for Lepidoptera.

A major goal of genome analysis is complete annotation, *i.e.*, a well-curated gene list. For the published assembly, consensus gene sets were predicted by gene finder programs such as BGF (Li *et al.* 2005) and GLEAN (Elsik *et al.* 2007) and auto-annotated in KAIKObase and SilkDB (Shimomura *et al.* 2009; Duan *et al.* 2010). However, the present silkworm genome assembly still contains inevitable gaps caused by enriched repetitive sequences and tightly clustered paralogous genes (The International Silkworm Genome Consortium 2008), which are the main sources of incorrect gene prediction. Another source of problems for gene prediction is the limited availability of full-length cDNA (FL-cDNA) sequences, which are needed to determine precise gene structures and ortholog-paralog relationships for comparative genomics. Distributing bioresources including FL-cDNA and cDNA clones to researchers is another mission of this project.

Here we report isolation and sequencing of 11,104 FL-cDNA clones chosen from 21 FL-cDNA libraries derived from 14 distinct silkworm tissues of different developmental stages. To determine a complete set of silkworm genes, we used 11,104 FL-cDNAs, 408,172 expressed sequence tags (ESTs), 2089 mRNA sequences registered in public databases, and 14,625 gene models as shown in Figure 1. We estimated the frequency of expression of a standard set of 16,823 genes, which were mapped on scaffolds and covered complete open reading frames culled from the FL-cDNA libraries and a large available EST database, and constructed a map of transcriptional expression for tissue-specific genes along the chromosomes. These data revealed distinctive patterns of tissue-specific and sexually dimorphic gene clusters. The extensive dataset we provide here should be valuable not only for studies in the silkworm but also for other insects.

**Figure 1:**
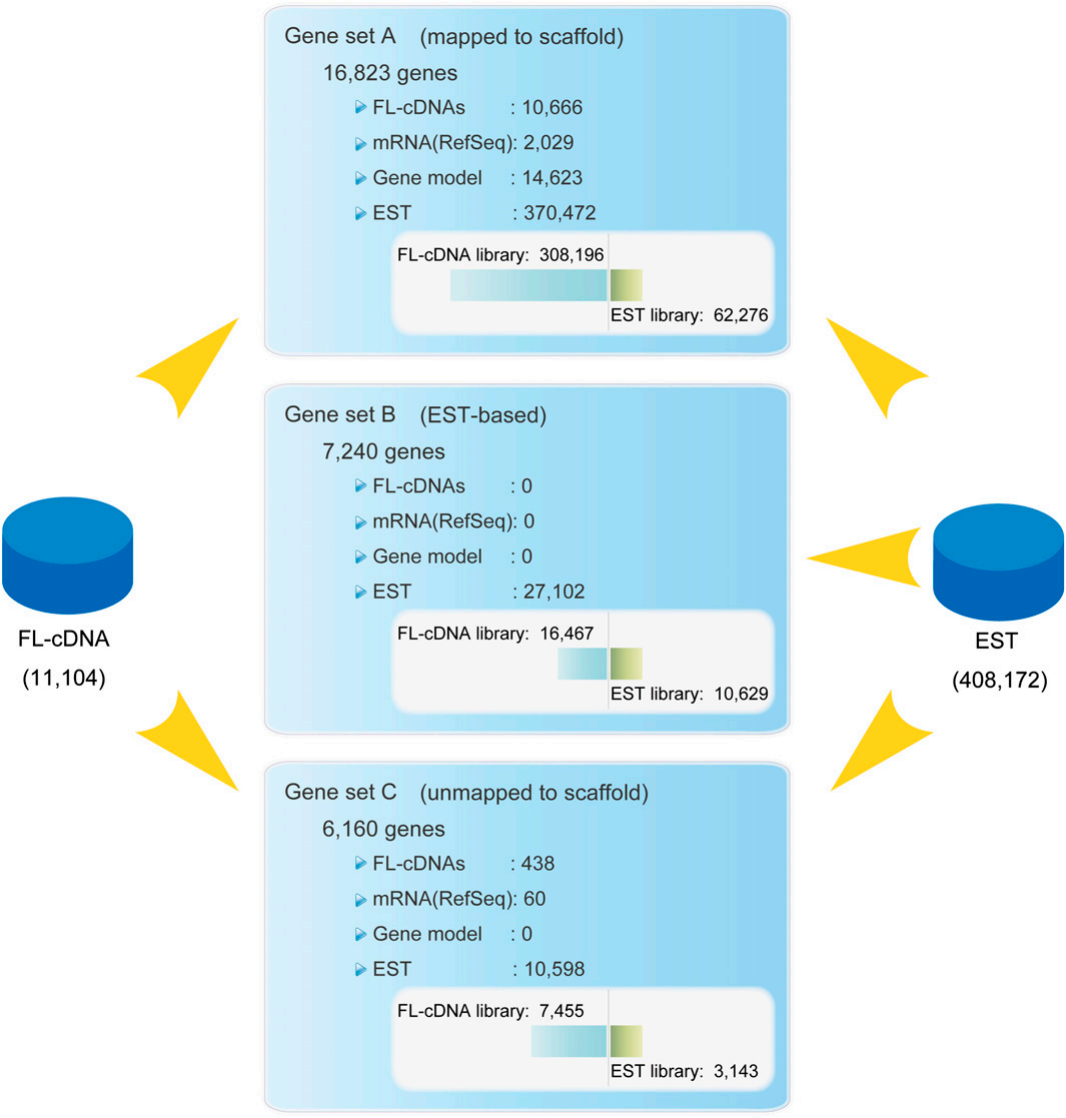
Overview of the gene-build for the silkworm genome. The white box in each gene set describes the composition of ESTs derived from FL-cDNA and cDNA libraries. ESTs used in Gene set B include ones derived from FL-cDNA libraries for which the full-length clone sequences were not yet determined.

## Materials and Methods

### Full-length cDNA library construction

Twenty-one FL-cDNA libraries were constructed from 14 distinct tissues of the domesticated silkworm, *B. mori*, at various developmental stages; details are listed in Table 1. Total RNA was extracted from various tissues dissected at appropriate stages of larvae, pupae, or adult moths and treated with TRIzol (Invitrogen) as described previously (Okamoto *et al.* 2008). FL-cDNA libraries were constructed with the Oligo-cap method performed by Toyobo Co. Ltd (Suzuki *et al.* 1997), the G-cap method by Hitachi Science Systems, Ltd., or the V-cap method by Hokkaido System Science Co. Ltd (Kato *et al.* 2005). More than 10,000 cDNA clones were picked up randomly from each cDNA library.

**Table 1:** Twenty-one full-length cDNA libraries analyzed

Name of full-length cDNA library	No. of Clones	Vector	Method (-cap)
famL (antenna & maxill. gl., larva)	14,044	pGCAP10	V-cap
fner (nerve system + brain, larva)	18,772	pGCAP10	V-cap
fcaL (corpora allata-cardiaca, larva)	17,222	pGCAP1	G-cap
fmxg (maxillary galea, larva)	4,366	pGCAP1	G-cap
fwd (wing disc)	11,515	pCMVFL	Oligo
fwgP (wing, pupa day 2−8)	17,521	pGCAP10	V-cap
ftes (testis, 5th larva)	16,487	pGCAP10	V-cap
bmte (testis, pupa day 4)	10,367	pGCAP10	V-cap
bmov (ovary, pupa day 4)	10,375	pGCAP10	V-cap
fmgV (midgut, 5th larva)	19,015	pGCAP10	V-cap
bmmt (malpighian tubule, larva)	10,104	pGCAP10	V-cap
fphe (pheromone gland, adult)	7,600	pGCAP1	G-cap
ffbm (male fat body, larva)	18,380	pGCAP10	V-cap
MFB (fat body, microbe-infected)	5,846	pGCAP1	G-cap
fepM (epidermis 4th molt)	6,588	pGCAP1	G-cap
fprW (prothoracic gland, W-stage)	6,000	pGCAP1	G-cap
fufe (unfertilized egg)	17,095	pGCAP10	V-cap
fdpe (diapaused egg)	5,714	pGCAP1	G-cap
e100 (embryo 100h)	5,025	pGCAP1	G-cap
fe8d (embryo day 8)	17,551	pGCAP10	V-cap
BmN (cultured cell)	8,543	pCMVFL	Oligo

Total: 21 full-length cDNA libraries Total: 248,130 clones.

### 5′ EST sequencing and determination of FL-cDNA clone candidates

Approximately 250,000 cDNA clones were randomly selected from 21 FL-cDNA libraries for determination of 5′ ESTs with the use of an ABI 3730 DNA sequencer. All 5′ ESTs were grouped into contigs or singletons under the criteria that if 5′ ESTs shared >95% identity over 100 consecutive bases for aligned regions, they were considered as identical clones transcribed from the same gene. The cDNA clone covering the most upstream region in a contig was chosen as a potential FL-cDNA candidate. All singletons were also potential candidates for FL-cDNAs. All candidate clones were then completely sequenced by a primer-walking method using an ABI 3730 DNA sequencer.

### Evaluation of FL-cDNA sequences

Vector sequences were filtered and low-quality bases (QV <20) were removed. The resulting FL-cDNA sequences were aligned with the silkworm genome assembly (The International Silkworm Genome Consortium 2008) to remove chimeric sequences. Perfect alignment with the genome assembly within 100 kb was used as a provisional criterion for absence of chimerism. The FL-cDNA sequences also were subjected to a BLAST search in the silkworm EST database (http://kaikocdna.dna.affrc.go.jp/) to determine whether mate-paired ESTs transcribed from the same gene could be aligned on the FL-cDNA sequence. If not, the FL-cDNA clone was considered likely to be chimeric or a splicing-isoform.

To facilitate the full sequencing of FL-cDNA candidates, the Illumina Genome Analyzer was used to obtain draft sequences of FL-cDNA clones >3 kb in size. The FL-cDNA candidates were divided into several sets, each containing 700 clones. Each set was then shotgun-sequenced using one lane of an Illumina Genome Analyzer flow cell. The reads were assembled by MuSICA as described in Kuroshu *et al.* (2010). We designed primer sets to confirm the assembled sequences by the primer-walking method with the ABI3730 sequencer.

### Alignment of FL-cDNAs onto the silkworm genome assembly

Genomic positions of FL-cDNAs were determined with est2genome, a software tool to aid the prediction of genes by sequence homology. est2genome is computationally expensive mainly due to usage of the Smith-Waterman algorithm (Smith and Waterman 1981) in return for higher precision. To reduce computation time, all FL-cDNAs were subjected to BLASTN search against silkworm genomic sequences prior to est2genome analysis to estimate their approximate genomic positions and filter out FL-cDNAs that were not aligned to a genomic sequence. We used a percent identity of 95% and alignment coverage of 0.5, the ratio of aligned length to total FL-cDNA length, as filtration criteria. If an FL-cDNA did not meet these criteria, it was classified as “unmapped” and discarded from further processing. We then cut out a subsequence of each FL-cDNA from a genomic sequence around the estimated genomic position for analysis by est2genome.

### Construction of an FL-cDNA database

Complete information for the FL-cDNA sequence data set was compiled into a database (http://sgp.dna.affrc.go.jp/FLcDNA/) with the following characteristics: clone name, full sequence, total size (bp), accession number, mapped scaffold, mapped chromosome number, chromosomal open reading frame location (start and stop codon positions), BLAST results in protein databases, and Gene Ontology (GO) and InterProScan terms, followed by identical EST numbers in each cDNA library.

### Expression profiles of FL-cDNA clones

Identical ESTs transcribed from each gene were collected by BLASTn search using criteria of >95% identity in a sequence >100 nucleotides in each cDNA library. Supporting Information, File S1 describes the frequency of identical ESTs in each tissue which includes not only FL-cDNA libraries but also cDNA libraries constructed by standard methods (Mita *et al.* 2003), yielding an expression profile of each FL-cDNA clone. Each FL-cDNA sequence was checked for identical ESTs in a deep database that consisted of ESTs from 21 FL-cDNA libraries, 36 previously published cDNA libraries (Mita *et al.* 2003), and 12 newly analyzed cDNA libraries (http://sgp.dna.affrc.go.jp/EST/page_pub.html), 408,172 ESTs in all. Using these expression profiles, we identified tissue-specific gene expression patterns for clones present in more than three copies with more than 90% tissue-specificity and compiled them in a “silkworm gene set” table (Table S1).

## Results and Discussion

### Characteristics of FL-cDNA sequences

A total of 248,130 5′ ESTs composed of at least 100 consecutive nucleotides with QV > 20 were obtained from 21 FL-cDNA libraries derived from 14 distinct tissues at different developmental stages of *B. mori* (Table 1) and assembled into 16,827 unique sequences (7124 singletons and 9703 contigs). The cDNA clone that covered the most upstream region of each contig was used as a representative of each group, *i.e.*, an FL-cDNA candidate clone, and was fully sequenced by the primer-walking method. We identified 11,104 FL-cDNA sequences (accession nos. AK377185-AK388575 in GenBank/EMBL/DDBJ), of which 438 could not be aligned on the silkworm genome assembly (Table 2). The mean length of the aligned FL-cDNAs was 1813 bp, and 10,838 cDNAs had open reading frames longer than 30 amino acids (Table 2). When the est2genome program (Mott 1997) in the EMBOSS (The European Molecular Biology Open Software Suite) package (Rice *et al.* 2000) was used, we found a total of 10,666 sequences (96.1%) were aligned at 9315 transcription sites. The whole data set, including improved annotations, was compiled into an FL-cDNA database (http://sgp.dna.affrc.go.jp/FLcDNA/).

**Table 2:** Basic statistics of *B. mori* FL cDNAs

Number of FL-cDNAs			11,104
Mean length, bp			1813
GC content			0.38
Maximum length, bp			10,430
Minimum length, bp			141
FL-cDNAs with CDS			10,838
FL-cDNAs with poly-A			8789
FL-cDNAs with CDS and poly-A			8789
Mapped FL-cDNAs		ORF +/−	Poly-A +/−
Mapped onto chromosomes	10,075	9,840/235	8,191/1,884
Scaffolds (unmapped on chromosomes)	591	569/22	485/106
Mapped total	10,666	10,409/257	8,676/1,990
Unmapped FL-cDNAs			438
Low coverage			380 (3.4%)
Low homology			58 (0.5%)
Mean alignment coverage			0.93
Mean percent identity			98.8

FL-cDNAs, full-length cDNAs; GC content, or guanine-cytosine content; CDS, coding sequence; ORF, open reading frame.

**Figure 2:**
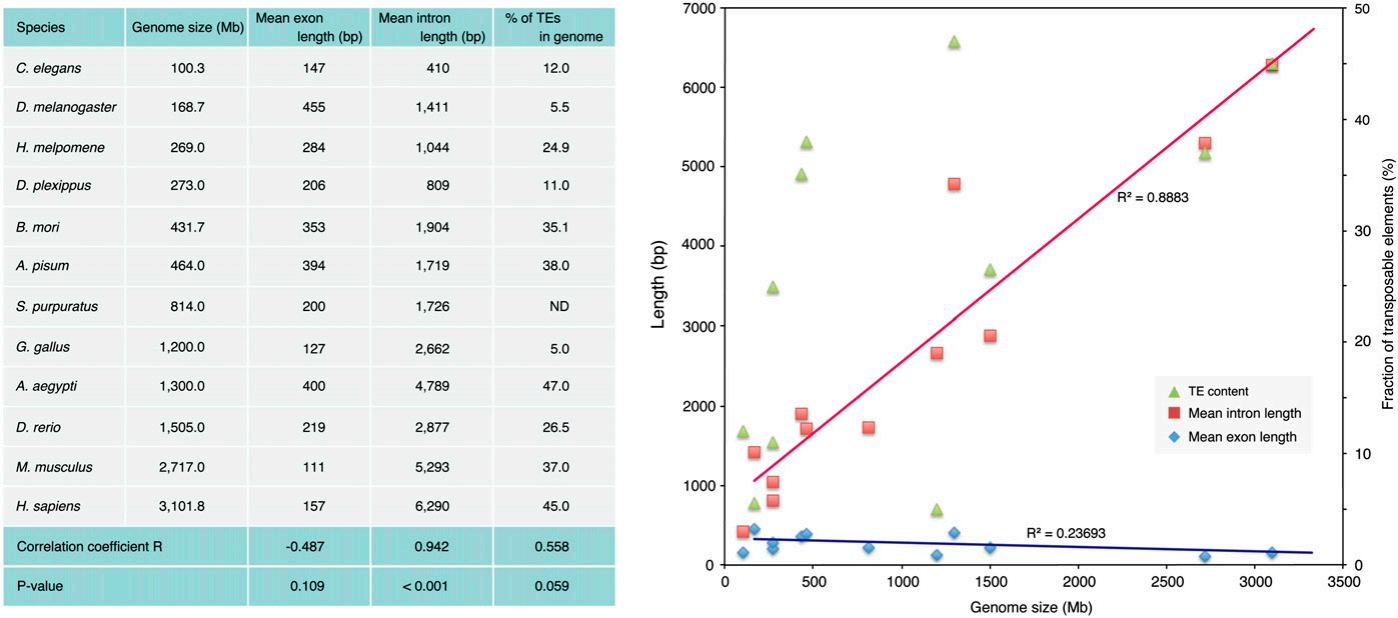
Relationships between genome size and average intron/exon lengths for various model species. To calculate the average intron and exon lengths for 11 species other than *B. mori* (*D. melanogaster*, *C. elegans*, *M. musculus*, *H. sapiens*, *D. plexippus*, *H. melpomene*, *G. gallus*, *A. aegypti*, *Acyrthosiphon pisum*, *Strongylocentrotus purpuratus*, and *Danio rerio*), the GTF-formatted gene annotation files were downloaded from the Ensembl ftp site ftp://ftp.ensembl.org and processed with a custom Perl script. Triangles denote the TE content.

By a comparison of 10,666 mapped FL-cDNA gene structures with previously reported silkworm gene prediction models (The International Silkworm Genome Consortium 2008) we found that 7504 FL-cDNAs matched with models, whereas 3162 (30%) showed no match. Among matched FL-cDNAs, our comparison showed that 1666 FL-cDNAs provided complete matches; however, 5059 structures comprising approximately three-fourths of the predicted genes were misannotated. As a comprehensive silkworm gene set (Table S1), previous annotations were updated by the use of available FL-cDNA data instead of predicted gene models and previous predicted gene models were used if there was no transcriptome data. By pair-wise comparison of transcripts mapped to a given locus, 2072 FL-cDNAs appeared to be derived from alternative splicing. The mean exon number per gene was 4.8, and mean exon and intron sizes were 353 bp and 1904 bp, respectively (Table 3). Comparison of these values relative to the genome size of 11 other model species showed a good correlation of the intron size with the genome size (Figure 2; R = 0.942; *P* < 0.001), indicating that the large introns of silkworm may have contributed to its relatively large genome size. We also compared the intron-genome size with the fraction of transposable elements in each genome (Figure 2), resulting in a rough correlation between genome size and TE content (R = 0.558; *P* < 0.059). The deviation in this value was considerably larger than the ratio of the mean intron size:genome size, which suggests that the large introns in the silkworm genome may have arisen in part from a high accumulation of repetitive sequences, mainly composed of transposons (Osanai-Futahashi *et al.* 2008). In contrast to the intron size, the average exon size had almost no correlation with the genome size (R = −0.487; *P* = 0.109), indicating there is very little variation in average exon length among the species examined.

**Table 3:** Characteristics of *B. mori* exons and introns

Exon	
Total number of exons	51,590
No of exons per transcript	
Mean	4.8
Median	4.0
Exon length, bp	
Max	8,783
Mean	353
Median	179
Min	27
Intron	
Intron length, bp	
Max	109,257
Mean	1904
Median	730
Min	34

#### Obtaining a comprehensive silkworm gene set:

To obtain a complete silkworm gene set, in addition to 11,104 FL-cDNA sequences, the available set of 408,172 ESTs, 2089 mRNA sequences in public databases, and 14,623 gene models predicted by GLEAN (Elsik *et al.* 2007) also were mapped and grouped. An overview of the procedures and results of this analysis is presented in Figure 1. FL-cDNA sequences, mRNA sequences in public databases, and 370,472 ESTs that could be aligned to FL-cDNAs or mRNAs in public databases and gene models were grouped into 16,823 gene sites (gene set A in Figure 1); these are compiled in Table S1A with gene IDs and used as the “silkworm gene set.” In addition, among 37,700 ESTs that did not align to gene set A, 27,102 ESTs could be mapped to the genome and were assembled into 7240 groups, which are referred to as “EST-based genes” (gene set B in Figure 1, sequences of which are available at http://sgp.dna.affrc.go.jp/FLcDNA/). It should be noted that gene set B includes ESTs derived from FL-cDNA libraries for which the full-length clone sequences have not yet been determined. The gene IDs for EST-based genes are prefixed with ‘e’ in Table S1B. Among them, 2268 sequences had open reading frames and were not annotated as TEs or viruses but may still be missing from gene set A of the silkworm genome assembly.

Gene set C is composed of the remaining 10,598 ESTs, 60 publicly available mRNAs, and 438 FL-cDNAs that were not aligned to the silkworm genome assembly. We grouped them into 6160 genes using CLOBB2 (http://www.nematodes.org/bioinformatics/CLOBB2/; Table S1C) whose sequences can be found at http://sgp.dna.affrc.go.jp/FLcDNA/). BLASTx search against the National Center for Biotechnology Information nonredundant database using a cutoff of 1e-10 was used to classify genes, indicating 2710 as “no hit” and 2612 genes as “other.” Of these 5322 genes, 3432 had open reading frames, which may have arisen from gap regions of the silkworm genome assembly. It should be noted that the silkworm genome assembly did not include the W chromosome because of its extremely high content of repetitive DNAs (Abe *et al.* 2005; The International Silkworm Genome Consortium 2008); however, transcripts derived from W might be included in cDNA libraries, which would be grouped in Gene set C. Taken together, we consider that the total number of silkworm genes may reach more than 20,000 (*e.g.*, 16,823 + 2268 + 3432 = 22,523).

### Silkworm FL-cDNA similarity comparison with other insect orders

Comparison of silkworm FL-cDNAs with gene sets of other insect orders with complete genome sequences provides an opportunity for exploring silkworm and Lepidoptera-specific genes. We compared the silkworm gene set with the gene sets of *Drosophila melanogaster* (Diptera), *Anopheles gambiae* (Diptera), *Aedes aegypti* (Diptera), *Culex quinquefasciatus* (Diptera), *Apis mellifera* (Hymenoptera), *Tribolium castaneum* (Coleoptera), *Acyrthosiphon pisum* (Homoptera), *Pediculus humanus* (Phthiraptera), *Danaus plexippus* (Lepidoptera), and *Heliconius melpomene* (Lepidoptera), 11 insect species of six orders in all (Table 4). For orthology analysis, in addition to the 11 insect species we used protein data sets of 142 noninsect species available in OrthoMCL DB (http://orthomcl.org/cgi-bin/OrthoMclWeb.cgi?rm=genome&type=summary).

**Table 4:** Protein datasets of 11 insect species tested for orthology using orthoMCL

Species	Order	No. Proteins	Data Source (URL)
*A. gambiae*	Diptera	12,457	http://orthomcl.org/cgi-bin/OrthoMclWeb.cgi?rm=genome&type=summary
*A. aegypti*	Diptera	15,419	http://orthomcl.org/cgi-bin/OrthoMclWeb.cgi?rm=genome&type=summay
*C. pipiens*	Diptera	18,883	http://orthomcl.org/cgi-bin/OrthoMclWeb.cgi?rm=genome&type=summary
*D. melanogaster*	Diptera	14,076	http://orthomcl.org/cgi-bin/OrthoMclWeb.cgi?rm=genome&type=summary
*A. mellifera*	Hymenoptera	9257	http://orthomcl.org/cgi-bin/OrthoMclWeb.cgi?rm=genome&type=summary
*A. pisum*	Hemiptera	10,466	http://orthomcl.org/cgi-bin/OrthoMclWeb.cgi?rm=genome&type=summary
*T. castaneum*	Coleoptera	16,645	http://beetlebase.org/
*P. humanus*	Phthiraptera	10,773	http://orthomcl.org/cgi-bin/OrthoMclWeb.cgi?rm=genome&type=summary
*B. mori*	Lepidoptera	22,163	http://sgp.dna.affrc.go.jp/KAIKObase/
*D. plexippus*	Lepidoptera	15,130	http://monarchbase.umassmed.edu
*H. melpomene*	Lepidoptera	12,829	http://butterflygenome.org/

**Figure 3:**
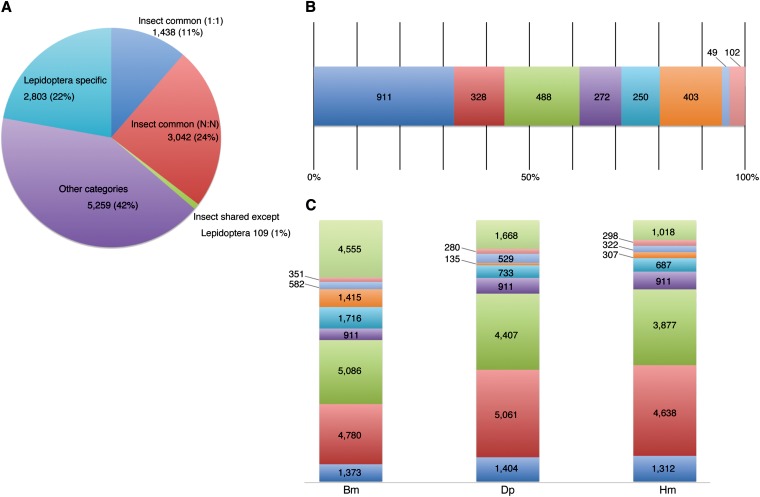
Orthology of 153 species including 11 insect species. (A) Pie chart: classification of 12,637 ortholog groups generated from protein datasets of 153 species, including 11 insect species listed in Table 4. 1:1 indicates universal single-copy genes. N:N indicates universal genes but with paralogs in at least one species. Numbers in the circles denote number of ortholog groups. (B) Bar chart: Orthology assignment of 2803 Lepidoptera specific ortholog groups. dark blue, Lepidoptera specific (1:1); red, Lepidoptera specific (N:N); green, Bm-Dp specific; purple, Bm-Hm specific; turquoise, Dp-Hm specific; orange, Bm specific; light blue, Dp specific; pink, Hm specific. (C) Classification of Lepidoptera-specific genes in three species. light green (top), not involved in ortholog groups; dark blue, common to 11 insect species (1:1); red, common to 11 insect species (N:N); green, others; purple, Lepidoptera specific (1:1); turquoise, Lepidoptera specific (N:N); orange, species specific; light blue, Bm-Dp specific for Bm, Dp-Bm specific for Dp, Hm-Bm specific for Hm; pink, Bm-Hm specific for Bm, Dp-Hm specific for Dp, Hm-Dp specific for Hm. Numbers in the boxes denote number of genes. Bm, *Bombyx mori*; Dp, *Danaus plexippus*; Hm, *Heliconius melpomene*.

Orthology analysis of protein data from 153 species including 11 insects using the program orthoMCL (Li *et al.* 2003) generated 12,637 ortholog groups, 4480 of which were designated as insect-shared, whereas 2803 were Lepidoptera-specific (Figure 3A). Figure 3B presents the classification of Lepidoptera-specific ortholog groups among three species. Silkworm showed the largest number of 403 silkworm-specific (Bm) ortholog groups compared with 49 assigned as Monarch-specific (Dp) and 102 as Heliconius-specific (Hm); the classification of Lepidoptera-specific genes is shown in Figure 3C.

To check for the presence of paralogs in each group, we compared the number of genes assigned to each ortholog group among the three Lepidoptera (Table 5). It is noteworthy that although the number of “insect-shared” genes and ortholog groups was almost the same among the three lepidopteran species, with relatively few paralogs (average ratio of 1.58 for #genes/#orthologs), the increase in number of “Lepidoptera-specific genes” and “Species-specific genes” was comparatively high. This finding might reflect an enhancement of species-specific proteins related to characteristic functions or traits that have evolved through gene-duplication events such as the large numbers of phototransduction-related genes in the monarch butterfly putatively involved in sensing skylight cues (Zhan *et al.* 2011) and chemosensory genes in the monarch and Heliconius butterflies used for host-recognition in feeding and oviposition (Zhan *et al.* 2011; The Heliconius Genome Consortium 2012).

**Table 5:** Comparison of orthologs and paralogs among three Lepidoptera

Species*^a^*	Insect-Common		Lep-Specific N:N		Species-Specific		
	No. Orthologs	No. Genes*^b^*	No. Orthologs	No. Genes*^b^*	No. Orthologs	No. Genes^b^	Ratios of No. Orthologs [Bm/ Dp, or Hm]
Bm	3042	4780 (1.57)	401	1917 (4.78)	531	1981 (3.73)	−
Dp	3042	5061 (1.66)	401	874 (2.18)	64	172 (2.69)	8.3 = [531/64]
Hm	3042	4638 (1.52)	401	771 (1.92)	41	97 (2.36)	13.0 = [531/41]

a Bm, *Bombyx mori*; Dp, *Danaus plexippus*; Hm, *Heliconius melpomene*.

b Parentheses denote the ratio of [No. Genes]/[No. Orthologs].

To our surprise, by our criteria silkworm had approximately 8- to 13-fold more silkworm-specific genes and an approximately 1.5-fold expansion of paralogs among the Lepidoptera-specific group compared with the other two species. These characteristics evoke a distinctive aspect of silkworm, its domestication and complete dependence on human care. Although we are still not clear what produced this phenomenon, a possible contributing factor may be an acceleration of evolution under artificial selection, which was reported to result in a significant diversity of carotenoid-binding protein genes responsible for cocoon color in the silkworm genome (Sakudoh *et al.* 2011). A complete genome comparison with *B. mandarina*, the wild ancestor of *B. mori*, will be of interest to test this hypothesis.

To understand the function of silkworm-specific proteins, we assigned 531 silkworm-specific genes with InterProScan and GO terms (Table S2) among which 147 groups were annotated with GO terms. We found a high abundance of genes associated with terms for protective immunity against microbial and viral pathogens such as *Moricin*, *Cecropin*, *Serpin*, *Lipoprotein*, *Glycoside hydrolase*, and *Guanylate-binding* (Tanaka *et al.* 2008), pheromone/hormone-related functions such as *fatty acid CoA reductase*, *Acyl CoA transferase*, *JH binding*, and *carboxylesterase*, and characteristic structural proteins such as *chorion* and *cuticle*. The fact that protective immunity-related genes were enriched in the silkworm-specific group together with expansion of those genes may again reflect artificial selection for disease-resistant and bacteria-resistant strains during domestication.

### Tissue-specifically expressed genes

Tissue-specific genes are of interest because their expression results in tissue-specific functions or traits. To identify them, each FL-cDNA sequence was checked for identical ESTs in the deep EST database and the number of identical ESTs for each FL-cDNA sequence across various tissues constituted its expression profile. If an FL-cDNA sequence with more than three identical EST clones had more than 90% identical ESTs derived from only one tissue in the EST database (*i.e.*, fewer than 10% of ESTs were derived from other tissues) we regarded the corresponding gene as tissue-specific. Using these criteria, we identified the tissue-specific genes by automated annotation; the integrated information is presented in Table S1. Table S3 summarizes the chromosomal distribution of tissue-specific genes in each tissue based on the genome alignment.

#### Comparison of gonad-specific genes reveals obvious sexual dimorphism:

Silkworm has a female heterogametic sex chromosome system with ZZ in males and ZW in females (Tanaka 1916; Goldsmith *et al.* 2005). An obvious difference in tissue-specific genes between ovary and testis was greater than 10-fold more testis-specific genes, 745, than the 68 ovary-specific genes; this finding is consistent with the observation that spermatogenesis is a much more complicated structural and developmental process than oogenesis. Similar observations have been made using EST microarray analysis (Xia *et al.* 2007). Mapping of testis-specific FL-cDNA sequences onto chromosomes yielded the same conclusions as previous work indicating that ch.Z (=ch.1) is enriched in testis-specific genes (Arunkumar *et al.* 2009).

**Figure 4:**
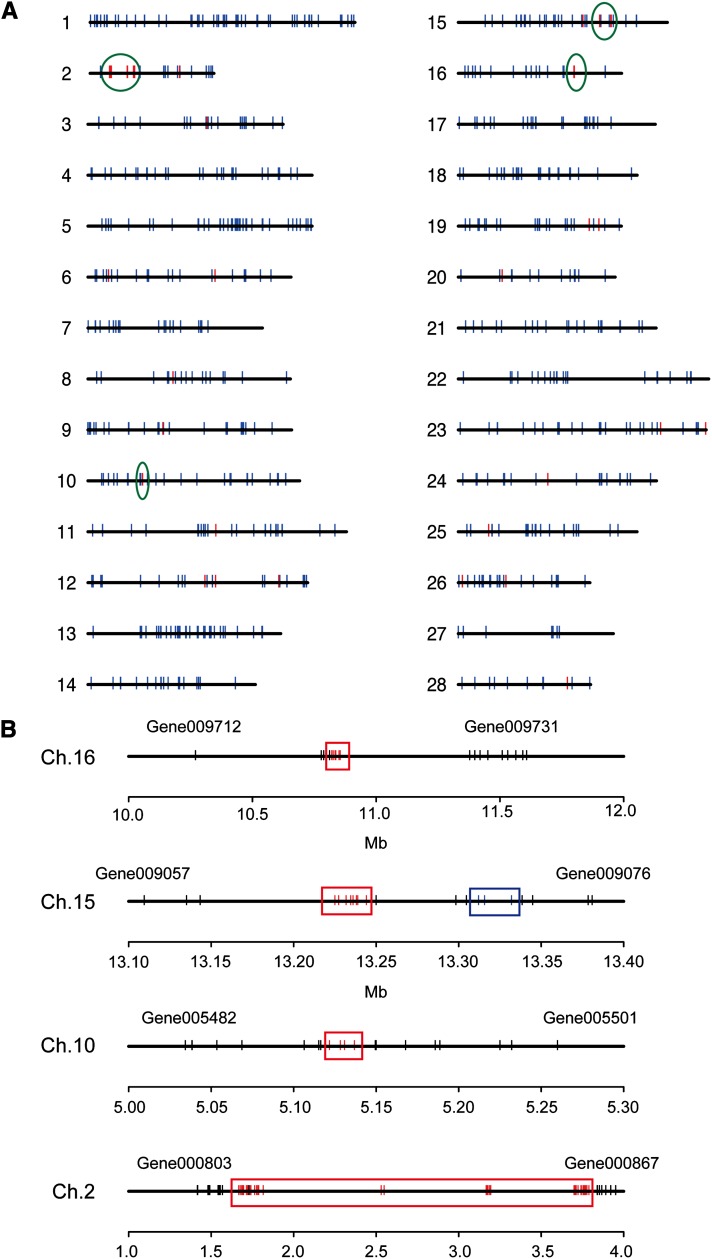
Sexual dimorphism in mapping of ovary-/testis-specific genes. (A) Chromosomal distribution of testis-specific (blue) and ovary-specific (red) genes. The ovary-specific gene clusters on ch.2, 10, 15, and 16 are circled in green and are presented with enlarged views in panel B. (B) Ovary-specific gene clusters on ch.2, 10, 15, and 16. Red bar, blue bar, and black bar denote ovary-specific gene, testis-specific gene, and nontissue-specific gene, respectively.

In contrast to testis-specific genes, the spatial distribution of ovary-specific genes provided a highly distinctive feature (Figure 4A). Seventy-four percent of the ovary-specific genes formed apparent gene clusters on only four chromosomes, ch.2, 10, 15, and 16 (Figure 4B). Among them, the largest was the chorion gene cluster on ch.2 in the region 1.78–3.79 Mb. We identified 27 chorion genes in this locus of the genome assembly (The International Silkworm Genome Consortium 2008); however, it has been reported that the chorion locus harbors more than 100 chorion genes (Eickbush and Izzo 2005). This discrepancy was probably caused by a tightly linked cluster of similar sequences, which made a large gap in the automated genome assembly. Just upstream of the chorion gene cluster, 12 ovary-specific genes (Gene000814, Gene000815, Gene000816, Gene000817, Gene000818, Gene000819, Gene000820, Gene000821, Gene000824, Gene000825, Gene000827, and Gene000828) with unknown function formed a cluster in a 100-kb region at 1.67–1.78 Mb. None of them showed significant homology with sequences in public protein databases, although their presence in the chorion region suggests they may contribute to oogenesis. On ch.10, four ovary-specific genes (Gene005491, Gene005493, Gene005494, and Gene005495) with unknown function formed a cluster in a 16-kb region. Eight ovary-specific genes, of which two genes seemed to encode an extensin 2-like protein and others were of unknown function, were located in a 20-kb region of ch.15 forming a tight gene cluster. In addition, an 85-kb region of ch.16 harbored six ovary-specific genes of unknown function. It is noteworthy that more than half of the ovary-specific genes were concentrated on ch.2 within a very narrow region of 2.1 Mb, which made a strong contrast with the dispersed distribution of testis-specific genes enriched on ch.Z. The testis-specific gene density on ch.Z was found to be 1.6 times greater than the average testis-specific gene density (Table S3), similar to the value of 1.75 times enrichment of testis-specific genes on ch.Z reported previously by Arunkumar *et al.* (2009). In addition, most other ovary-specific genes like the chorion genes formed bigger clustered gene families with overlapping functions than testis-specific genes (Table 6). This distinctive nature might be crucial for oogenesis whereby oocytes which are very large compared to spermatocytes must complete development in a relatively short time.

**Table 6:** Fractions of clustered tissue-specific genes

Tissue	No. Tissue-Specific Genes	No. Clustered Tissue-Specific Genes	Fraction in Cluster, %	Total No. of Tissue-Specific Gene Clusters	Fraction of Genes Duplicated in Clusters (No. Genes)	Tissue-Characteristic Proteins Expressed By Duplicated Genes in Clusters
Brain	36	9	25	4	0.22 (2)	
Compound eyes	6	0	0	0	—	
Corpora allata-cardiaca complex	52	8	15	4	0.25 (2)	Adipokinetic hormone
Maxillary galea	1	0	0	0	—	
Wing	67	30	45	9	0.57 (17)	Cuticular protein/osiris
Wing disc	54	8	15	4	0.25 (2)	Paralytic peptide binding protein
Testis	745	327	44	113	0.20 (66)	Carboxypeptidase/ beta-tubulin/ pyruvate kinase
Ovary	68*^a^*	53*^a^*	78	8	0.45 (24)*^a^*	Chorion
Midgut	185	88	48	27	0.89 (78)	Carboxyesterase/ lipase/glucosidase/30kP protease/fatty acid-binding protein/trypsin/cuticle/SEC14/ Bm 122
Malpighian tubule	48	11	23	4	1.0 (11)	Sugar transporter/ Na^+^-dependent transporter/synaptic vesicle transporter/adenylate cyclase
Pheromone gland	14	6	43	3	0.67 (4)	Fatty-acid reductase/aldehyde oxidase
Silkgland	22	4	18	2	1.0 (4)	Sericin-like
Fat body	13	5	38	2	0.6 (3)	30-kDa protein
Epidermis	26	17	63	6	0.82 (14)	Cuticular protein
Verson’s gland	11	3	27	1	1.0 (3)	Serpin
Total	1,365	569	42	187	0.40 (230)	

a These numbers are low estimates since many chorion genes were known to be missing from the assembly (Eickbush and Izzo 2005).

Superimposing the chromosomal distribution images of testis-specific and ovary-specific genes revealed dramatic features of sexual dimorphism (Figure 4A). Ch.Z, where testis-specific genes were enriched, completely lacked ovary-specific genes, whereas testis-specific genes were entirely missing from the ovary-specific gene cluster region of ch.2:1.67–3.79 Mb. Testis-specific genes were also lacking or exclusively depleted in the other ovary-specific gene cluster regions on Chs. 10, 15, and 16. The ovary-specific gene regions were well separated from the testis-specific, gene-enriched regions, which may enable efficient gene expression in each type of gonad. For example, ovary-specific gene cluster regions may use a euchromatic structure that leads to efficient gene expression in ovaries, whereas testis-specific genes may be localized in heterochromatic domains for effective repression of expression in ovaries, and *vice versa* in testes. Analyses of PIWI-interacting RNAs on ovary-specific regions support the establishment of large euchromatic domains for their expression in ovary (File S1; Figure S1).

#### The osiris gene cluster conserves a characteristic structure and expression profile across insects:

**Figure 5:**
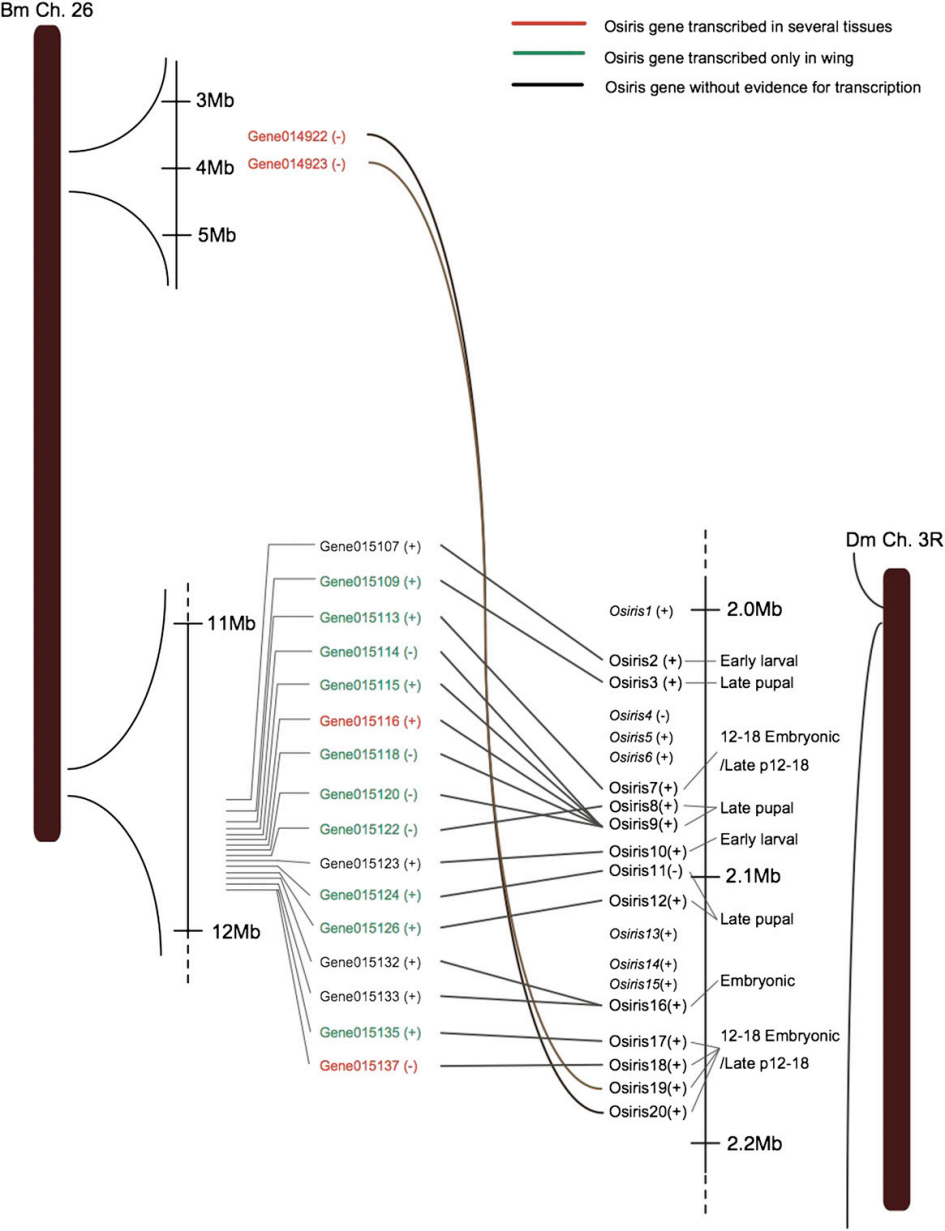
Synteny of the *osiris* gene cluster between silkworm and fruitfly. Green, *osiris* genes transcribed only in wing; red, *osiris* genes transcribed in several tissues; black, *osiris* genes with no evidence for transcription. Silkworm *osiris* genes are numbered according to the *Drosophila* gene set; (+) and (–) denote the orientation of the gene.

*Osiris* genes are highly conserved and clustered in insects; however, their function is still unknown (Dorer *et al.* 2003; Shah *et al.* 2012). Interestingly, we found several wing-specific *osiris* genes in *B. mori* that were clustered on ch.26. Among them we could identify several *Drosophila osiris* homologs, although some of them were missing from the silkworm genome, including *Osi1*, *Osi4-6*, *Osi13-15*, and *Osi23*. It was reported that *Drosophila osiris* genes encode a novel family of transmembrane proteins (Dorer *et al.* 2003), all of which contain a well-conserved pair of cysteine amino acid residues. Using the protein structure prediction programs PSORT (Nakai and Horton 1999) and SOSUI (Hirokawa *et al.* 1998), we found silkworm *osiris* proteins had the same structural features. *Bmosi2* and *Bmosi16* provided no evidence for transcription and may be pseudogenes or misannotated. Although we found no silkworm homologs to *Drosophila osiris* genes 4−6 and 13−15, the remainder of *Bmosi3*–*Bmosi18* were clustered in a single locus of ch.26:11,546,404-11,956,406 in the same order as *Drosophila osiris* genes *1-20* (Figure 5; Table S4). In addition, five copies of *Bmosi9* were found in a cluster between *Bmosi7* and *Bmosi8* in the region ch26:11,684,158-11,776,381. By phylogenetic analysis, these 5 copies were formed by gene duplication events after separation of Lepidoptera from Diptera. The homologs of *Dmosi21* and *22* were unlinked to the main silkworm *osiris* gene cluster on ch. 26, which was consistent with their independent genetic linkage mapping on ch.4 and ch.12, respectively. These results indicate an explicit microsynteny between silkworm and fruitfly genomes (Figure 5).

*Bmosi3*–*Bmosi12* showed wing-specific expression except for *osi9-3*, which was epidermis-specific, and *Bmosi10*, which had no hit in the EST database. *Bmosi18*, which was located at the very end of the cluster, was also not wing-specific. *Bmosi19* and *Bmosi20*, which were located in a single site near the other end of ch.26 in the reverse order, were also not wing-specific, similar to *Bmosi18*. *Bmosi3–Bmosi12* in the main gene cluster were conserved to have wing-specific expression in the pupal stage, similar to corresponding homologs in *Drosophila*, which show peak expression at the late pupal stage. *Bmosi18–20* had a different expression profile, suggesting that the original *osiris* gene cluster was partitioned into two parts with different timing of gene expression. Subsequently, the *osi19*/*osi20* region was translocated from its position in the original gene cluster near the end of ch.26 to the other end of the same chromosome during silkworm evolution. The *osiris* gene cluster provides another example suggesting that the same tissue-specific genes may be clustered to form a tissue-specific chromatin domain. The largest cuticular protein gene cluster on ch.22 reinforces this observation (File S1; Figure S2).

#### Characteristic expression profile of a 30-kDa lipoprotein gene cluster correlates with a distinct function:

**Figure 6:**
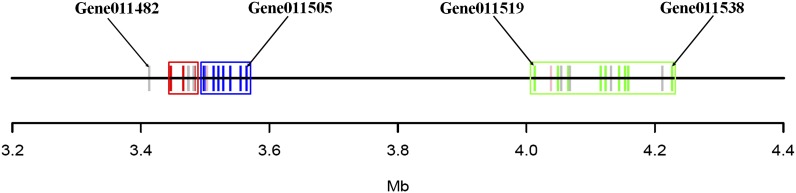
Map of thirty-three 30-kDa protein genes forming a cluster on chromosome 20. In the first gene cluster composed of nineteen 30-kDa protein genes, four fat body-specific genes and one MT-specific 30kDa protein gene (red) are located in the former half of the cluster, whereas nine 30-kDa protein genes mainly expressed in brain-nervous system (blue) occupy the latter half of the cluster. Six genes showed no hit in the EST database (gray). A second 30-kDa protein gene cluster composed of 14 genes was located 440 kb from the former gene cluster. Nine of fourteen genes were specifically expressed in an embryonic stage (green), whereas one was mainly expressed in fat body; the remaining four genes showed no hit in the EST db.

Biosynthesis of the Lepidoptera-specific 30-kDa lipoprotein gene family whose function is not fully understood occurs in a stage-dependent fashion in fat body (Ujita *et al.* 2002). We found thirty-three 30-kD a protein genes localized in an 820-kb region of ch.20 forming two gene clusters (Figure 6, Table S5). The first gene cluster in the region spanning 3,412,956−3,565,568 harbored nineteen 30-kDa protein genes; four of these were fat body-specific and one was malpighian tubule (MT)-specific. Interestingly, whereas three of the 19 genes in the cluster seemed not to express, nine genes were transcribed extensively and primarily in the brain-nervous system. The expression levels of these nine genes were significantly greater than in fat body (Table S5), indicating the brain-nervous system is a major contributor of 30-kDa protein transcripts. In addition, the four fat body and MT-specific genes were localized in the 5′ portion of the gene cluster, whereas the nine genes expressed mainly in brain-nervous system occupied the other half of the cluster (Figure 6). The second gene cluster spanning the region 4,012,903−4,227,867 was composed of fourteen 30-kDa protein genes, nine of which expressed exclusively or mainly at an embryonic stage (Figure 6; Table S5). These observations suggest an as yet-unknown function for 30-kDa proteins that has not been reported previously and further support the idea of tissue-specific chromosome domains.

### Tissue-specific gene clusters

Mapping of tissue-specific genes onto chromosomes is informative for understanding the relationship between chromosome structure and transcription. As can be clearly seen in ovary (Figure 4B), wing (Figure 5; Figure S2) and fat body (Figure 6), tissue-specific genes showed a tendency to be located close to one another in clusters. In Table 6, we summarize the fraction of tissue-specific genes clustering in each tissue whereby we recognized two genes expressed in a given tissue as belonging to a single cluster if they were within 100 kb on the same chromosome. The traditional model describes the higher-order structure of interphase chromosomes as formed by a series of loops of about 50−150 kb DNA that are attached to a peripheral lamina or other internal structures, such as scaffolds or skeletons (Cook 1995; Mishra and Karch 1999; Maeshima and Laemmli 2003). Moreover, large-scale chromatin loops greater than 1 Mb have been observed in G1 phase chromosomes of human cells (Yokota *et al.* 1995) and inducible loci of mammalian cells (Hu *et al.* 2009). Thus, we used the criterion of “within 100 kb” for a cluster search of 1365 tissue-specific genes on the 475-Mb silkworm genome. Table 6 shows that, on average, 42% of tissue-specific genes were in clusters, although the fraction varied from 0.15 to 0.78 among tissues. We checked whether the clustering of tissue-specific genes indeed occurs in silkworm by invoking the Fisher exact test (Routlege 2005) to test the null hypothesis for independence of the type of gene and the type of genes adjacent to it. The test rejected the null hypothesis (*P* = 2.2e-16), indicating that tissue-specific genes have a tendency of forming clusters (Table S6).

To consider the driving factors for clustering, it is highly informative to check the fraction of tissue-specific genes in clusters formed by gene duplication events, since gene duplication will overwhelmingly produce new gene copies in very close proximity to the original gene locus which are likely to retain their tissue specificity unless they evolve new functions. The rates of gene duplication are summarized in Table 6, showing a deep dependence on tissue. For differentiated tissues such as wing, midgut, MT, pheromone gland, silk gland, fat body, and epidermis, a very high rate of duplication and clustering of tissue-specific genes was observed, which suggests that the respective tissue produces these characteristic proteins or enzymes at high levels. On the contrary, in ovary, only the chorion gene cluster was formed by gene duplication, and the other gene clusters on ch.10, 15, and 16 were composed of different kinds of genes. The same tendency was observed in testis-specific gene clusters, where the rate of gene duplication was considerably less. Even for differentiated tissues such as wing, midgut, and epidermis, some of the clustered tissue-specific genes were not derived from gene duplication events.

### FL-cDNA clones and sequence data as bioresources

All FL-cDNA sequence data were incorporated into KAIKObase and can be downloaded from its associated GBrowse. The silkworm gene sets in Table S1 combined with the results of orthology analysis were also integrated into KAIKObase. In addition, a comprehensive Silkworm Genome Annotation Project is in progress by the Silkworm Genome Annotation Consortium using the complete FL-cDNA data. All FL-cDNA and standard cDNA clones are maintained at the National Institute of Agrobiological Sciences and the University of Tokyo and are available on request (see KAIKObase). The distribution of the DNA clones is also supported by the National Bioresource Project (NBRP).

The sequences obtained in this study are critical for more accurate genome annotation of this lepidopteran model insect, enhancing genomic and functional studies of Lepidoptera and comparative analyses of *B. mori* with other insect orders, and yielding new insights into gene evolution and the existence of lepidopteran-specific genes. Additionally, the data presented here including the organization and expression of testis- and ovary-specific genes, *osiris*, 30K, and cuticular protein genes (File S1; Table 6) show a clustering tendency for tissue-specific genes in the silkworm genome and extend earlier observations of striking differences in the organization of sexually dimorphic sets of genes.

## Supplementary Material

Supporting Information
