# Supplementary material for: Large Scale Full-Length cDNA Sequencing Reveals a Unique Genomic Landscape in a Lepidopteran Model Insect, Bombyx mori
Source: G3 (Bethesda). 2013 Sep 1;3(9):1481–92. doi: 10.1534/g3.113.006239 (PMC3755909; doi:10.1534/g3.113.006239)
Supplement: Supporting Information [file supp_3_9_1481__index.html]

Large Scale Full-Length cDNA Sequencing Reveals a Unique Genomic Landscape in a Lepidopteran Model Insect, Bombyx mori — Supporting Information 

# Large Scale Full-Length cDNA Sequencing Reveals a Unique Genomic Landscape in a Lepidopteran Model Insect, *Bombyx mori*

## Supporting Information for Suetsugu *et al.*, 2013

**Files in this Data Supplement:**

- Supporting Information - Figures S1-S2, File S1, and Tables S1-S6 (PDF, 518 KB)
- Figure S1 - Density plots of ovarian piRNAs in the ovary-specific gene regions and a representative large ovarian piRNA cluster (PDF, 341 KB)
- Figure S2 - The largest cuticular protein gene cluster on ch.22 (PDF, 79 KB)
- File S1 - Negative regulation of ovary-specific gene regions by piRNA (PDF, 119 KB)
- Table S3 - Summary of the chromosomal distribution of tissue-specific genes in each tissue (PDF, 96 KB)
- Table S4 - Mapping of *Bm osiris* genes (PDF, 77 KB)
- Table S6 - Result of Fisher's Exact Test to evaluate the association between type of a gene and type of its adjacent genes (PDF, 67 KB)
- Table S1 - A dataset table of silkworm gene sets presented in Figure 3: (A) Silkworm Gene Set A, (B) Silkworm Gene Set B, and (C) Silkworm Gene Set C. (.xlsx, 5 MB)
- Table S2 - Annotated ortholog groups of 531 silkworm-specific orthologs with InterProScan and GO-terms (.xlsx, 74 KB)
- Table S5 - 30kDa protein genes forming a gene cluster on chromosome 20 (.xls, 43 KB)
