# Supplementary material for: Large Scale Full-Length cDNA Sequencing Reveals a Unique Genomic Landscape in a Lepidopteran Model Insect, Bombyx mori
Source: G3 (Bethesda). 2013 Sep 1;3(9):1481–92. doi: 10.1534/g3.113.006239 (PMC3755909; doi:10.1534/g3.113.006239)
Supplement: Supporting Information [file supp_g3.113.006239_FigureS1.pdf]

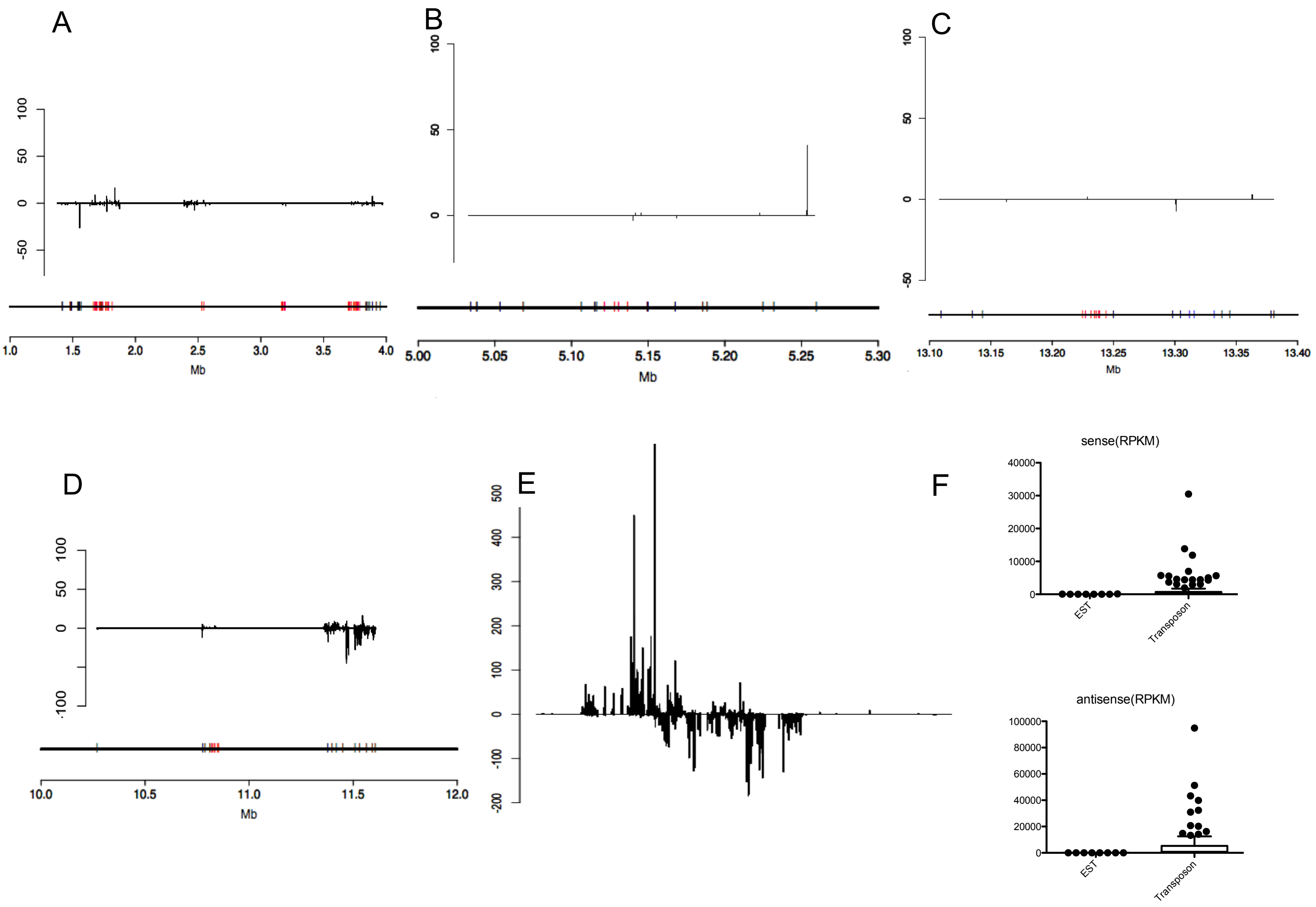

Figure S1 Density plots of ovarian piRNAs in the ovary-specific gene regions and a representative large ovarian piRNA cluster. Each bar represents the relative abundance of piRNAs that were uniquely mapped to the silkworm genome (unique mappers). (A-D) Ovary-specific gene clusters of ch.2 (A), ch.10 (B), ch.15 (C) and ch.16 (D). Upper line denotes ovarian piRNA level. The positions of genes are shown by vertical lines in the lower line, where ovary-specific genes are colored red. (E) A representative large ovarian piRNA cluster in the region ch.14:2,547,598-2,847,509. (F) Box plots show relative abundance of sense and antisense piRNAs mapped to 47 ovary-specific ESTs or 121 transposons. Relative abundance of piRNAs matching each element is expressed as reads per million per a kilobase (RPKM).
