# Supplementary material for: Large Scale Full-Length cDNA Sequencing Reveals a Unique Genomic Landscape in a Lepidopteran Model Insect, Bombyx mori
Source: G3 (Bethesda). 2013 Sep 1;3(9):1481–92. doi: 10.1534/g3.113.006239 (PMC3755909; doi:10.1534/g3.113.006239)
Supplement: Supporting Information [file supp_g3.113.006239_FigureS2.pdf]

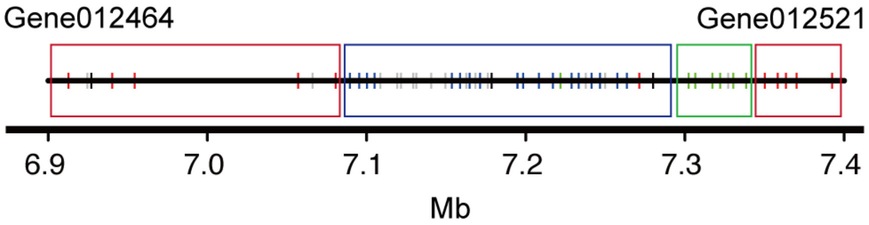

Figure S2 The largest cuticular protein gene cluster on ch.22. The largest cuticular protein gene cluster is divided into 4 parts. Both ends are comprised of wing-specific genes (red), whereas the two central parts containing more than 40 cuticular protein genes are larval undifferentiated disc-specific (blue) and embryonic stage-specific (green).
