# Supplementary material for: Large Scale Full-Length cDNA Sequencing Reveals a Unique Genomic Landscape in a Lepidopteran Model Insect, Bombyx mori
Source: G3 (Bethesda). 2013 Sep 1;3(9):1481–92. doi: 10.1534/g3.113.006239 (PMC3755909; doi:10.1534/g3.113.006239)
Supplement: Supporting Information [file supp_g3.113.006239_FileS1.pdf]

File S1

Negative regulation of ovary-specific gene regions by piRNA

To address whether the ovary specific gene regions were euchromatic, we investigated the genomic mapping pattern of PIWI-interacting RNAs (piRNAs). piRNAs are small RNAs of 23-30 nucleotides in length which act with PIWI subfamily proteins in animal gonads to silence transposon activity (Aravin *et al.* 2007; Ghildiyal and Zamore 2009; Malone and Hannon 2009). piRNAs are often complementary to transposons and thus are capable of helping PIWI proteins cleave transposon mRNAs. Genomic mapping typically shows a striking enrichment in dispersed genomic loci called piRNA clusters spanning several to hundreds of kilobases which often contain transposon related-sequences. Previous studies have implicated piRNAs in heterochromatin formation. In *Drosophila*, piRNA clusters tend to be heterochromatic and mutations in piRNA pathway genes cause chromatin opening (Brennecke *et al.* 2007; Huisinga and Elgin 2009; Klenov *et al.* 2007). Silkworm piRNA clusters are likely to be heterochromatic as well (Kawaoka *et al.* 2009). In these regards, piRNA clusters are a reliable indicator for transposon contents and chromatin states in animal gonads. Thus, we visualized the genomic mapping patterns of previously reported silkworm ovarian piRNAs (Kawaoka *et al.* 2011) in the ovary-specific gene regions and found them to be depleted (Figure S1A-D). In parallel, mapping of ovarian piRNAs to mRNAs derived from these regions revealed a much lower level in comparison with piRNAs that mapped to transposons (Figure S1E and F). Collectively, our analyses revealed a mutually exclusive relationship between the ovary specific gene regions and ovarian piRNAs, supporting the view that the ovary-specific gene regions establish large euchromatic domains in the ovarian genome.

For piRNA-related analysis, we used a previously reported ovarian piRNA library (Kawaoka *et al.* 2011). Ovarian piRNA reads were mapped to the silkworm genome and ESTs as described previously (Kawaoka *et al.* 2011). Normalized piRNA reads were expressed as read per million. For visualizing genome-mapping of ovarian piRNAs, we focused exclusively on unique mappers (Brennecke *et al.* 2007; Kawaoka *et al.* 2008a). R-code and in-house UNIX programs used for this analysis will be provided upon request to S. Kawaoka, kawaoka@ss.ab.a.u-tokyo.ac.jp.

The Z chromosome is defeminized in silkworm

The sex chromosomes, apart from their primary role in sex determination, are also involved in sexual dimorphism (Iyengar *et al.* 2002; Rice 1984), and their genetic landscape has undergone distinctive changes in the course of evolution compared to autosomes. Interesting findings on the organization of genes with sex-limited expression have emerged from the analysis and mapping of 11,104 FL-cDNAs in the silkworm. We found that the Z chromosome is conspicuous by the absence of any female-enhanced or female-biased genes; in other words, it is heavily defeminized. In contrast, it is enriched in male-specific and male-biased genes as reported previously (Arunkumar *et al.* 2009; Zhao *et al.* 2011).

Recent studies suggest that similarities and dissimilarities exist within and between male and female heterogametic systems (Ellegren 2011). For example, the chicken Z chromosome is known to harbor a massive

tandem array of testis-specific genes (Bellott *et al.* 2010), and clustering of testis-specific genes is also observed on human (Ross *et al.* 2005) and mouse (Mueller *et al.* 2008) X chromosomes. However, although the present analysis revealed clusters of ovary-specific genes on autosomes, no clusters of tandemly duplicated testis-specific genes were found on the *B. mori* Z chromosome or autosomes.

Female-biased genes located on the Z chromosome are underrepresented among those expressed in the germline of the chicken. This may result from the inactivation of sex chromosomes during female meiosis, referred to as female MSCI (Iyengar *et al.* 2002), which was first reported in this species (Schoenmakers *et al.* 2009). Although the presence of MSCI has not been reported in silkworm, we cannot rule out the existence of such a phenomenon. We therefore speculate that the observed depletion of ovary-specific genes on the silkworm Z chromosome may result in part because of silencing of sex chromosomes during female meiosis due to MSCI.

Unlike many animal species where male heterogamety prevails (e.g., mammals and dipterans), *B. mori* has a female heterogametic sex chromosome system. Silkworm also lacks sex chromosome dosage compensation (Zha *et al.* 2009). This makes the Z chromosome a favorable place for male advantageous genes, as genes on Z are expressed in a double dose in males, which also carry 2/3 of the Z chromosomes present in a population (Rice 1984). Therefore, it is possible that the observed depletion of female-enhanced genes on the Z may be preferred in part because of disadvantages experienced in females from their hemizygous condition and an absence of dosage compensation.

Characteristics of wing-specific genes involved in wing differentiation

Studying the change of gene expression during differentiation by comparing wing disc and wing specific genes can reveal tissue-specific patterns in the recruitment of gene sets at different stages of development. Fifty-four silkworm genes were regarded as a wing disc-specific gene set, whereas 67 genes were wing-specific. Among the wing disc-specific genes, a chromodomain-helicase chd1 homolog (Gene002825, ch.5), son of sevenless protein (Gene008822, ch.15), and Msx2-interacting protein (Gene001413, ch.3) genes were expressed at a high level, suggesting they may be involved in cell differentiation functions (Newberry *et al.* 1999; Gaspar-Maia *et al.* 2009; Pierre *et al.* 2011). Also a gene homologous to *discs large1* (Gene007132, ch.12) which is involved in neural differentiation and cellular growth control during larval development (Wood and Bryant 1991) was specifically transcribed in wing-disc.

During wing development, a large quantity of various types of cuticular proteins is required for wing and scale formation. Eighteen cuticle protein genes were identified to be expressed specifically in wing, among which 8 genes contained an RR-1 motif, 5 genes had an RR-2 motif and the remaining 5 genes contained glycine-rich repeats (Willis *et al.* 2005; Futahashi *et al.* 2008). Cuticular protein genes are reported to be present in gene clusters in insects including *Bombyx* (Futahashi *et al.* 2008), *Drosophila* (Karouzou *et al.* 2007), *Anopheles* (Cornman *et al.* 2008) and *Tribolium* (Tribolium Genome Sequence Consortium 2008). The wing-specific cuticular protein genes identified in the present study seemed to have the same tendency. Four wing-specific cuticular protein genes, *BmorCPG39* (Gene010482), *BmorCPH42* (Gene010483), *BmorCPG46* (Gene010484) and *BmorCPG38* (Gene010486), were clustered

in a region of approximately 100kb of ch.18. Analysis of the expression level of the largest cluster of 56 cuticular protein genes in the 6,918,803-7,403,507 region of ch.22 suggested that it is divided into 4 sections related to expression profile (Figure S2). Both ends were enriched or composed of wing-specific cuticular protein genes. The ch.22:6,918,803-7,090,619 region involved 3 wing-specific genes and a cuticular protein gene mainly expressed in wing, though one testis-specific cuticular protein gene was also found in this section. In contrast, the central 2 portions of the largest gene cluster, ch22:7,015,600-7,329,298, which harbored more than 40 cuticular protein genes, consisted of genes mainly expressed in wing-discs, larval antenna, compound eye discs and late embryos (i.e., genes expressed mainly in undifferentiated larval discs, in addition to embryonic stage-specific genes). Among the 40 cuticular protein genes, some of them apparently were produced by gene duplication events; however, the remaining genes appeared to be homologs of distinct *Drosophila* orthologs based on molecular phylogeny (Futahashi *et al.* 2008). This pattern of organization is consistent with other findings indicating a higher level mechanism for stage- and tissue-specific activation of euchromatin .

Comparison of silkgland-specific genes and the PSG-MSG transcriptome

The major components of silk are fibroin and sericin, which are synthesized and secreted in the silkgland. The posterior part of the silkgland (PSG) exclusively synthesizes the fibroin proteins including fibroin heavy-chain, fibroin light-chain and fibrohexamerin P25, whereas the middle part of the silkgland (MSG) produces the coat or glue protein sericins. We found 5 PSG-specific genes, including the fibroin H-chain gene (ch.25), fibroin L-chain gene (ch.14) and Bmhexamerin P25 gene (ch.2). The other two PSG-specific genes were Gene014431 and Gene014504 which encode transmembrane emp24 and ecdysteroid UDP-glycosyl-transferase, respectively. Transmembrane emp24 may be responsible for secretion of synthesized fibroin proteins, and ecdysteroid UDP-glycosyl transferase is related to regulation of ecdysone activation, which may regulate the timing of fibroin gene expression.

In contrast, we mapped 12 MSG-specific genes including 3 sericin genes, *Ser1, Ser2* and *Ser3* (Couble *et al.* 1987; Takasu *et al.* 2007). The sericin genes were located within 2 Mb on ch.11, where 3 other MSG-specific genes (Gene006229, Gene006235 and Gene006284) were found. Two of them which contain a rather high content of serine residues are likely homologs of sericin protein. Thus, this sericin locus involves 6 MSG-specific genes. Two alcohol dehydrogenase genes (Gene003058, Gene003062) tandemly aligned on ch. 5 were MSG-specific. The MSG-specific gene, Gene002828, encoding sulfotransferase was also on this chromosome. One of 6 Juvenile Hormone (JH) acid methyl transferase genes (Gene007113) clustered on ch.12 was MSG-specific and may participate in regulation of sericin gene expression in the MSG (The International Silkworm Genome Consortium 2008). Two other MSG-specific genes (Gene013782 and Gene014649) on ch.24 and ch.25 were unknown in function. Two MSG-specific serine protease inhibitor genes (Gene016373, Gene016374) corresponding to serpin 18 and serpin 16 formed a cluster in unmapped Bm_scaf322 (Zou *et al.* 2009). A gene encoding a cysteine-rich trypsin inhibitor on ch.6, Gene003545, was shared by both PSG/MSG; it and the two serpins likely have antibacterial activity and play an important role in protecting silk proteins.

It was noticeable that more abundant MSG-specific genes were transcribed in the MSG than in the PSG, which may reflect the various functions and complex structures of the coat proteins responsible for protection of core silk filaments and efficient spinning of fibers (Takasu *et al.* 2007). The present results for the silkgland are supported by a recent SAGE analysis of PSG and MSG transcriptomes (Royer *et al.* 2011) reporting that more highly abundant tags derived from non-silk protein mRNAs are distributed exclusively in the MSG library.

Tissue-specific genes in other tissues

Tissue-specific genes mainly contribute to the physiological functions and traits of the corresponding tissues. For example, the corpora allata exclusively synthesize JH. A whole suite of JH biosynthesis pathway associated genes was found in silkworm genome assemblies (The International Silkworm Genome Consortium 2008). The genes encoding adipokinetic prohormone types 1 and 2 (Gene004134 and Gene004135), located close to each other near the end of ch.7 to form a cluster, were found to be exclusively expressed in corpora allata-corpora cardiaca complex. These genes serve to stimulate the release of energy sources to flight muscle (Noyes and Schaffer 1990; Roller *et al.* 2008), although silkmoths cannot fly.

Brain is responsible for nerve pathways and neuropeptide metabolism. We found 36 brain-specific genes such as those encoding two sodium/glucose symporters, protease, acetylcholine-esterase, FMRF amide neuropeptide, and DCN5-related N-acetyltransferase neuroendocrine molecules. Three zinc finger protein genes (Gene000540, Gene000542 and Gene000547) formed a cluster in an approximately 50kb region:15,685,942-15,733,749 of ch. Z. It is interesting that the most highly expressed brain-specific gene was the lipocalin family gene *Bombyrin,* which is found in the central nervous system (Sakai *et al.* 2001; Sanchez *et al.* 2000). One lipocalin protein of grasshopper was reported to play a role in axon guidance (Sanchez *et al.* 2000). Taken together, the evidence suggests this protein may be involved in maintenance of the silkworm ganglia system or behavior.

The midgut serves as a main organ where digestive enzyme synthesis and secretion, as well as nutrient digestion and absorption, take place. We found 185 midgut-specific genes. Ten lipase genes were midgut-specific, among which 7 genes were associated in midgut-specific gene clusters (Gene007037, Gene007038, Gene007218, Gene007216, Gene012011, Gene012012 and Gene012013). We also found 10 midgut-specific *trypsin* genes and 12 *chymotrypsin* genes. Four of the *trypsin* genes (Gene002990-Gene002993) formed a tight cluster in a 55kb region of ch. 5, 6 midgut-specific chymotrypsin genes were clustered within 260kb at 1,399,970-1,656,103 of ch.16, and 3 chymotrypsin genes (Gene010680-Gene010682) formed a tight cluster in a 6kb region of ch.18. Additional peptidase genes, including *a-* and *b-glucosidase*, *serine carboxypeptidase*, *metalloprotease*, and *amylase*, were specifically expressed in midgut. Six *aminopeptidase* genes formed a cluster at the end of ch.9 (Gene004805, Gene004806 and Gene004807-Gene004810). Five *lipase* genes, one *trypsin* gene and one *serine carboxypeptidase* gene seemed to form a broad cluster spanning 10 Mb on ch.12, and 14 midgut-specific genes (Gene011223, Gene011224, Gene011228, Gene011241-Gene011251) with unknown function made a tight gene cluster spanning 411 kb on ch.19. In all, 88 of 185 midgut-specific genes corresponding to 48% formed gene clusters (Table 6).

The malpighian tubule (MT) is the excretory organ in insects. We found 50 MT-specific genes, among which we identified 16 transporter genes associated with ion, lipid, sugar and other substrates. Ten of 16 MT-specific genes (Gene003246, Gene003250, Gene003251; Gene004204, AK387086, Gene004205, Gene004206; Gene012848, Gene012850, Gene012855) formed 3 gene clusters near the end regions of ch.6, ch.8 and ch.22. It is interesting that 22 out of 47 MT-specific genes corresponding to 47% were located within 2.5 Mb of the ends of chromosomes, whereas in other tissues 25-40% of tissue-specific genes were localized near the ends of chromosomes. We speculate that this tendency may arise from a specialized chromatin structure. For example, chromatin in MT cells has been reported to display a granular appearance in codling moth (Fukova *et al.* 2007), suggesting that MT-specific genes may be associated with a largely heterochromatic structure that interferes with transcription. Thus, localization of MT-specific genes near the ends of chromatin might allow their expression to be possible and efficient under these conditions.

The female pheromone gland (PG), where we found 14 PG-specific genes (Gene001488, Gene006820, Gene006971, Gene006972, Gene008931, Gene010523, Gene010524, Gene011129, Gene012599, Gene012638, Gene012955, Gene013258, Gene015899 and Gene013466), exclusively produces pheromone. Two key genes associated with pheromone biosynthesis, *fatty-acid reductase* and *acyl-CoA delta-II desaturase* (Moto *et al.* 2003), were expressed in PG specifically. In addition, two lipase genes (Gene001488 and Gene010523) related to lipid metabolic processes were expressed in PG. One larval cuticular protein gene was also expressed specifically in PG, which may be responsible for the exclusive synthesis of PG segments.

A relatively small number of 13 genes (Gene003146, Gene003156, Gene003580, Gene004254, Gene007913, Gene011486, Gene011487, Gene0011492, Gene013545, Gene014309, Gene015103, Gene015629, and Gene015681) were expressed specifically in fat body; their function is centered in immune-response and storage such as the 30kDa proteins used for metabolism during pupal development. We identified three kinds of antibacterial protein genes, including *cecropin A*, *protease inhibitor-like protein* and 3 *gloverins* (Kawaoka *et al.* 2008b), which formed a broad cluster spanning 2.1 Mb on ch.28.

We found a cluster composed of five serpin genes (Gene001648, Gene001649, Gene001650, Gene001652 and Gene001653 corresponding to serpins 15, 17, 24, 20 and 25, respectively) in the 100kb region 17,728,552-17,828,627 of ch.3; three of them (Gene001648, Gene001649 and Gene001652) were Verson's gland-specific, whereas the other two seemed not to express (data not shown). A published phylogenetic analysis of insect serpin genes (Zou *et al.* 2009) showed that these five genes form a silkworm-specific clade, indicating recent gene duplications. Serpins are a superfamily of protease inhibitors, most of which regulate protease-mediated processes in diverse tissues. The finding of a cluster of Verson's gland serpin genes provides insight into their function and an example of tissue-specific chromosome domains.

LITERATURE CITED in File S1

Aravin, A. A., G. J. Hannon, and J. Brennecke, 2007 The Piwi-piRNA pathway provides an adaptive defense in the transposon arms race. Science **318**:761-764.

Arunkumar, K. P., K. Mita, and J. Nagaraju, 2009 Silkworm Z chromosome is enriched in testis-specific genes. Genetics **182**:493-501.

Bellott, D. W., H. Skaletsky, T. Pyntikova, E. R. Mardis, T. Graves, *et al.*, 2010 Convergent evolution of chicken Z and human X chromosomes by expansion and gene acquisition. Nature **466**:612-616.

Brennecke, J., A. A. Aravin, A. Stark, M. Dus, M. Kellis, *et al.*, 2007 Discrete small RNA-generating loci as master regulators of transposon activity in Drosophila. Cell **128**:1089–1103.

Cornman, R. S., T. Togawa, W. A. Dunn, W. A. Dunn, N. He, *et al.*, 2008 Annotation and analysis of a large cuticular protein family with the R&R consensus in *Anopheles gambiae*. BMC Genomics **9**:22.

Couble, P., J. J. Michaille, A. Garel, M. L. Couble ML, and J. C. Prudhomme, 1987 Developmental switches of sericin mRNA splicing in individual cells of *Bombyx mori* silkgland. Dev Biol **124**:431–440.

Ellegren, H., 2011 Sex-chromosome evolution: recent progress and the influence of male and female heterogamety. Nature rev Genetics **12**:157-166.

Fukova, I., W. Traut, M. Vitkova, P. Nguyen, S. Kubickova, *et al.*, 2007 Probing the W chromosome of the codling moth, *Cydia pomonella*, with sequences from microdissected sex chromatin. Chromosoma **116**:135-145.

Futahashi, R., S. Okamoto, H. Kawasaki, Y. Zhong, M. Iwanaga, *et al.*, 2008 Genome-wide identification of cuticular protein genes in the silkworm, *Bombyx mori*. Insect Biochem Mol Biol **38**:1138-1146.

Gaspar-Maia, A., A. Alajem, F. Polesso, R. Sridharan, M. J. Mason, *et al.*, 2009 Chd1 regulates open chromatin and pluripotency of embryonic stem cells. Nature **460**:863-868.

Ghildiyal, M., and P. D. Zamore, 2009 Small silencing RNAs: an expanding universe. Nat Rev Genet **10**:94-108.

Huisinga, K. L., and S. C. Elgin, 2009 Small RNA-directed heterochromatin formation in the context of development: what flies might learn from fission yeast. Biochim Biophys Acta **1789**:3-16.

Iyengar, V. K., H. K. Reeve, and T. Eisner, 2002 Paternal inheritance of a female moth's mating preference. Nature **419**:830-832.

Karouzou, M. V., Y. Spyropoulos, V. A. Iconomidou, R. S. Cornman, S. J. Hamodrakas, *et al.*, 2007 Drosophila cuticular proteins with the R&R consensus annotation and classification with a new tool for discriminating RR-1 and RR-2 sequences. Insect Biochem Mol Biol **37**:754-760.

Kawaoka, S., K. Kadota, Y. Arai, Y. Suzuki, T. Fujii, *et al.*, 2011 The silkworm W chromosome is a source of female-enriched piRNAs. RNA **12**:2144-2151.

Kawaoka, S., N. Hayashi, S. Katsuma, H. Kishino, Y. Kohara, *et al.*, 2008a Bombyx small RNAs: Genomic defense system against transposons in the silkworm, *Bombyx mori*. Insect Biochem Mol Biol **38**:1058-1065.

Kawaoka, S., N. Hayashi, Y. Suzuki, H. Abe, S. Sugano, *et al.*, 2009 The Bombyx ovary-derived cell line endogenously expresses PIWI/PIWI-interacting RNA complexes. RNA **15**:1258-1264.

Kawaoka, S., S. Katsuma, T. Daimon, R. Isono, N. Omuro, *et al.*, 2008b Functional analysis of four Gloverin-like genes in the silkworm, *Bombyx mori*. Arch Insect Biochem Physiol **67**:87-96.

Klenov, M. S., S. A. Lavrov, A. D. Stolyarenko, S. S. Ryazansky, A. A. Aravin, *et al.*, 2007 Repeat-associated siRNAs cause chromatin silencing of retrotransposons in the *Drosophila melanogaster* germline. Nucleic Acids Res **35**:5430-5438.

Malone, C. D., and G. J. Hannon, 2009 Molecular evolution of piRNA and transposon control pathways in *Drosophila*. Cold Spring Harb Symp Quant Biol 74:225-234.

Moto, K., T. Yoshiga, M. Yamamoto, S. Takahashi, K. Okano, *et al.*, 2003 Pheromone gland-specific fatty-acyl reductase of the silkmoth, *Bombyx mori*. Proc Natl Acad Sci USA **100**:9156-9161.

Mueller, J. L., S. K. Mahadevaiah, P. J. Park, P. E. Warburton, D. C. Page, *et al.*, 2008 The mouse X chromosome is enriched for multicopy testis genes showing postmeiotic expression. Nature genet **40**:794-799.

Newberry, E. P., T. Latifi, and D. A. Towler, 1999 The RRM domain of MINT, a novel Msx2 binding protein, recognizes and regulates the rat osteocalcin promoter. Biochemistry **38**:10678-10690.

Noyes, B. E., and M. H. Schaffer, 1990 The structurally similar neuropeptides adipokinetic hormone I and II are derived from similar very small mRNAs. J Biol Chem **265**:483-489.

Pierre, S., A. S. Bats, A. Chevallier, L. C. Bui, A. Ambolet-Camoit, *et al.*, 2011 Induction of the Ras activator Son of Sevenless 1 by environmental pollutants mediates their effects on cellular proliferation. Biochem Pharmacol **81**:304-313.

Rice, W. R., 1984 Sex chromosomes and the evolution of sexual di-morphism. Evolution **38**:735–742.

Roller, L., N. Yamanaka, K. Watanabe, I. Daubnerova, D. Zitnan, *et al.*, 2008 The unique evolution of neuropeptide genes in the silkworm *Bombyx mori*. Insect Biochem Mol Biol **38**:1147-1157.

Ross, M. T., D. V. Grafham, A. J. Coffey, S. Scherer, K. McLay, *et al.*, 2005 The DNA sequence of the human X chromosome. Nature **434**:325-37.

Royer, C., J. Briolay, A. Garel, P. Brouilly, S. Sasanuma, *et al.*, 2011 Novel genes differentially expressed between posterior and median silk gland identified by SAGE-aided transcriptome analysis. Insect Biochem Mol Biol **41**:118-124.

Sakai, M., C. Wu, and K. Suzuki, 2001 Nucleotide and deduced amino acid sequence of a cDNA encoding a lipocalin protein in the central nervous system of *Bombyx mori*. Sericology **70**:105-111.

Sanchez, D., M. D. Ganfornina, and M. J. Bastiani, 2000 Lazarillo, a neuronal lipocalin in grasshopper with a role in axon guidance. Biochim Biophys Acta **1482**:102-109.

Schoenmakers, S., E. Wassenaar, J. W. Hoogerbrugge, J. S. E. Laven, J. A. Grootegoed, *et al.*, 2009 Female meiotic sex chromosome inactivation in chicken. PLoS genet **5**:e1000466.

Takasu, Y., H. Yamada, T. Tamura, H. Sezutsu, K. Mita, *et al.*, 2007 Identification and characterization of a novel sericin gene expressed in the anterior middle silk gland of the silkworm *Bombyx mori*. Insect Biochem Mol Biol **37**:1234-1240.

The International Silkworm Genome Consortium, 2008 The genome of a lepidopteran model insect, the silkworm *Bombyx mori*. Insect Biochem Mol Biol **38**:1036-1045.

Tribolium Genome Sequence Consortium, 2008 The genome of the model beetles and pest *Tribolium castaneum*. Nature **452**:949-955.

Willis, J. H., V. A. Iconomidou, R. F. Smith, and S. J. Hamodrasks, 2005 Cuticular proteins. In: Gilbert LI, Iatrou K, Gill SS (Eds), *Comparative Molecular Insect Science*. vol. 4. Elsevier pp. 79-110.

Wood, D. F., and P. J. Bryant, 1991 The *discs-large* tumor suppressor gene Drosophila encodes a guanylate kinase homolog localized at separate junctions. Cell **66**:451-464.

Zha, X., Q. Xia, J. Duan, C. Wang, N. He, *et al.*, 2009 Dosage analysis of Z chromosome genes using microarray in silkworm. Insect Biochem Mol Biol **39**:315–321.

Zhao, M., X-F. Zha, J. Liu, W-J. Zhang, N-J. He, *et al.*, 2011 Global expression profile of silkworm genes from larval to pupal stages: Toward a comprehensive understanding of sexual differences. Insect Sci **18**:607-618.

Zou, Z., Z. Picheng, H. Weng, K. Mita, and H. Jiang, 2009 A comparative analysis of serpin genes in the silkworm genome. Genomics **93**:367-375.
