# Supplementary material for: Large Scale Full-Length cDNA Sequencing Reveals a Unique Genomic Landscape in a Lepidopteran Model Insect, Bombyx mori
Source: G3 (Bethesda). 2013 Sep 1;3(9):1481–92. doi: 10.1534/g3.113.006239 (PMC3755909; doi:10.1534/g3.113.006239)
Supplement: Supporting Information [file supp_g3.113.006239_TableS3.pdf]

Table S3 Summary of the chromosomal distribution of tissue-specific genes in each tissue.

Chromosome No.	1(Z)	2	3	4	5	6	7	8	9	10	11	12	13	14	15	16	17	18	19	20	21	22	23	24	25	26	27	28
**antenna**	0	0	0	0	0	0	0	0	0	0	3	1	1	0	0	3	0	1	6	0	0	0	0	0	0	1	0	1
**brain**	5	0	4	0	2	4	1	1	1	0	1	4	0	2	0	1	1	0	0	2	0	1	2	1	1	0	0	0
**compound eyes**	0	0	0	2	0	0	0	0	0	0	0	1	0	0	0	0	0	0	0	0	1	1	0	0	0	0	1	0
**corpora allata**	5	2	2	2	2	2	3	1	1	3	3	3	1	0	1	2	1	1	4	0	1	3	4	2	0	1	0	0
**maxillary galea**	0	0	0	0	0	0	0	0	0	0	0	0	0	0	0	0	0	0	1	0	0	0	0	0	0	0	0	0
**wing**	1	0	1	3	1	2	2	1	0	0	2	1	0	2	2	1	1	7	3	1	3	10	6	2	2	7	1	2
**wing disc**	5	3	1	3	1	1	2	0	1	3	5	2	0	1	2	3	0	1	2	2	1	1	1	4	3	0	0	4
**testis**	57	15	17	28	44	22	28	23	28	25	22	19	45	21	29	18	28	25	28	12	25	23	28	23	28	20	11	9
**ovary**	0	33	0	0	0	1	0	0	1	4	0	2	0	0	10	6	0	0	3	1	0	0	3	1	0	1	0	1
**midgut**	2	3	4	8	11	5	4	2	10	4	8	7	8	6	10	12	3	8	16	12	10	2	6	3	5	4	0	1
**malpighian tubule**	1	1	4	0	1	6	1	5	2	4	2	0	2	1	1	1	2	0	0	1	2	3	1	1	1	0	3	0
**pheromone gland**	0	0	1	0	0	0	0	0	0	0	1	2	0	0	1	0	0	2	1	0	0	2	2	0	0	0	0	0
**silkgland**	0	1	0	0	2	1	1	0	0	1	4	1	0	0	0	0	0	1	0	0	0	0	1	1	3	0	0	0
**fat body**	0	0	0	0	2	1	0	1	0	0	0	0	1	0	0	0	0	0	0	3	0	0	1	0	1	1	0	2
**epidermis**	0	0	0	0	3	1	1	1	0	0	2	0	0	0	0	0	0	4	0	0	0	5	1	1	0	1	0	0
**Verson's gland**	0	0	0	0	0	0	0	0	0	0	0	0	0	0	0	0	0	0	0	0	0	0	0	0	0	0	0	0
**prothoracic gland**	0	0	0	0	0	0	0	0	0	0	0	0	0	0	0	0	0	0	0	0	0	0	0	0	0	0	0	0
**embryo**	0	0	0	0	0	0	0	0	0	0	0	0	0	0	0	0	0	0	0	0	0	0	0	0	0	0	0	0
**cell**	0	0	0	0	0	0	0	0	0	0	0	0	0	0	0	0	0	0	0	0	0	0	0	0	0	0	0	0

In silkworm, chromosome 1 is assigned to sex chromosome Z.
