# Supplementary material for: Large Scale Full-Length cDNA Sequencing Reveals a Unique Genomic Landscape in a Lepidopteran Model Insect, Bombyx mori
Source: G3 (Bethesda). 2013 Sep 1;3(9):1481–92. doi: 10.1534/g3.113.006239 (PMC3755909; doi:10.1534/g3.113.006239)
Supplement: Supporting Information [file supp_g3.113.006239_TableS4.pdf]

Table S4 Mapping of *Bm osiris* genes.

Name	Local gene ID	Chr.	Start	End	BmOsi expression
Bmosi2	Gene015107	26	11,546,404	11,546,832	No hit in EST
Bmosi3	Gene015109	26	11,575,601	11,598,597	Wing-specific
Bmosi7	Gene015113	26	11,671,958	11,672,992	Wing-specific
Bmosi9-1	Gene015114	26	11,684,158	11,693,923	MSG/Wing-main
Bmosi9-2	Gene015115	26	11,719,076	11,724,428	Wing-main
Bmosi9-3	Gene015116	26	11,734,389	11,739,610	Epidermis-specific
Bmosi9-4	Gene015118	26	11,750,726	11,757,781	Wing-specific
Bmosi9-5	Gene015120	26	11,769,930	11,776,381	Wing-specific
Bmosi8	Gene015122	26	11,781,935	11,791,287	Wing-specific
Bmosi10	Gene015123	26	11,802,171	11,808,845	No hit in EST
Bmosi11	Gene015124	26	11,813,011	11,814,641	Wing-specific
Bmosi12	Gene015126	26	11,823,241	11,841,238	Wing-specific
Bmosi16-1	Gene015132	26	11,878,923	11,883,393	No hit in EST
Bmosi16-2	Gene015133	26	11,887,539	11,890,619	No hit in EST
Bmosi17	Gene015135	26	11,907,284	11,950,051	Wing-specific
Bmosi18	Gene015137	26	11,955,247	11,956,406	Epidermis-main
Bmosi19	Gene014922	26	3,999,143	4,000,555	Epidermis-main
Bmosi20	Gene014923	26	3,989,935	3,991,185	Ovary-main
Bmosi21	Gene002044	4	10,844,311	10,845,881	Wing-specific
Bmosi22	Gene007289	12	12,127,331	12,132,618	Wing-specific
