# Supplementary material for: Large Scale Full-Length cDNA Sequencing Reveals a Unique Genomic Landscape in a Lepidopteran Model Insect, Bombyx mori
Source: G3 (Bethesda). 2013 Sep 1;3(9):1481–92. doi: 10.1534/g3.113.006239 (PMC3755909; doi:10.1534/g3.113.006239)
Supplement: Supporting Information [file supp_g3.113.006239_TableS6.pdf]

Table S6 Result of Fisher's Exact Test to evaluate the association between type of a gene and type of its adjacent genes.

	Adjacent gene on at least one side is tissue specific	Adjacent genes on both sides are non tissue specific
Gene is tissue specific	424	858
Gene is non-tissue specific	1,945	12,602

A one-tailed Fisher's Exact Test (Routlege 2005) was performed with a level of significance of $P < 0.01$ to evaluate the association between the type of a gene and the type of its adjacent genes. The result was P=2.2e-16, *i.e.*, the null hypothesis can be rejected. There was a significant difference in the ratio of the type of adjacent genes between tissue specific and non-tissue specific genes, indicating a higher probability that a tissue-specific gene resides next to another tissue-specific gene than a non tissue-specific gene; *i.e.*, tissue-specific genes have a tendency to be clustered.
